# The Population Pharmacokinetics of Meropenem in Adult Patients With Rifampicin-Sensitive Pulmonary Tuberculosis

**DOI:** 10.3389/fphar.2021.637618

**Published:** 2021-06-29

**Authors:** Ahmed A. Abulfathi, Veronique de Jager, Elana van Brakel, Helmuth Reuter, Nikhil Gupte, Naadira Vanker, Grace L. Barnes, Eric Nuermberger, Susan E. Dorman, Andreas H. Diacon, Kelly E. Dooley, Elin M. Svensson

**Affiliations:** ^1^ Division of Clinical Pharmacology, Department of Medicine, Faculty of Medicine and Health Sciences, Stellenbosch University, South Africa; ^2^ Department of Clinical Pharmacology and Therapeutics, College of Medical Sciences, University of Maiduguri, Maiduguri, Nigeria; ^3^ Task Applied Science, Bellville, South Africa; ^4^ Department of Medicine, Center for Tuberculosis Research, Johns Hopkins University, Baltimore, MD, United States; ^5^ Department of Medicine, Medical University of South Carolina, Charleston, SC, United States; ^6^ Department of Medicine, Faculty of Medicine and Health Sciences, Stellenbosch University, South Africa; ^7^ Divisions of Clinical Pharmacology and Infectious Diseases, Department of Medicine, Johns Hopkins University Center for Tuberculosis Research, Baltimore, MD, United States; ^8^ Department of Pharmaceutical Biosciences, Uppsala University, Uppsala, Sweden; ^9^ Department of Pharmacy, Radboud Institute for Health Sciences, Radboud University Medical Center, Nijmegen, Netherlands

**Keywords:** meropenem, population pharmacokinetic (PK) model, tuberculosis, pharmacokinetics analysis, drug sensitive TB

## Abstract

**Background:** Meropenem is being investigated for repurposing as an anti-tuberculosis drug. This study aimed to develop a meropenem population pharmacokinetics model in patients with pulmonary *tuberculosis* and identify covariates explaining inter-individual variability.

**Methods:** Patients were randomized to one of four treatment groups: meropenem 2 g three times daily plus oral rifampicin 20 mg/kg once daily, meropenem 2 g three times daily, meropenem 1 g three times daily, and meropenem 3 g once daily. Meropenem was administered by intravenous infusion over 0.5–1 h. All patients also received oral amoxicillin/clavulanate together with each meropenem dose, and treatments continued daily for 14 days. Intensive plasma pharmacokinetics sampling over 8 h was conducted on the 14th day of the study. Nonlinear mixed-effects modeling was used for data analysis. The best model was chosen based on likelihood metrics, goodness-of-fit plots, and parsimony. Covariates were tested stepwise.

**Results:** A total of 404 concentration measurements from 49 patients were included in the analysis. A two-compartment model parameterized with clearance (CL), inter-compartmental clearance (Q), and central (V1) and peripheral (V2) volumes of distribution fitted the data well. Typical values of CL, Q, V1, and V2 were 11.8 L/h, 3.26 L/h, 14.2 L, and 3.12 L, respectively. The relative standard errors of the parameter estimates ranged from 3.8 to 35.4%. The covariate relations included in the final model were creatinine clearance on CL and allometric scaling with body weight on all disposition parameters. An effect of age on CL as previously reported could not be identified.

**Conclusion:** A two-compartment model described meropenem population pharmacokinetics in patients with pulmonary *tuberculosis* well. Covariates found to improve model fit were creatinine clearance and body weight but not rifampicin treatment. The final model will be used for an integrated pharmacokinetics/pharmacodynamics analysis linking meropenem exposure to early bactericidal activity.

## Introduction

The epidemic rise in multi-drug resistant (MDR) and extensively drug resistant *tuberculosis* (XDR-TB) threatens the progress made in reducing morbidity, mortality, and efforts in *tuberculosis* eradication (WHO Guideline, 2019). Medicines included in the current World Health Organization’s guideline for MDR- and XDR-TB treatment may be inaccessible in resource-limited settings and many older second-line anti-tuberculosis agents have significant toxicity, some of which could be life threatening and/or irreversible (WHO Guideline, 2019). Drug repurposing or the optimized use of existing drugs or combination of drugs is a cheaper alternative to the development of new chemical entities and could accelerate the process of finding good alternative treatments.

*Mycobacterium tuberculosis* is historically considered resistant to β-lactam antibiotics including carbapenems because of the constitutive production of a broad-spectrum β-lactamase called BlaC (Solapure et al., 2013; van Rijn et al., 2019). The addition of a β-lactamase inhibitor such as clavulanate prevents BlaC-mediated breakdown of β-lactams (van Rijn et al., 2019). Furthermore, meropenem is both a poor substrate and inhibitor of BlaC, thus, administering meropenem together with clavulanate is an attractive combination (Hugonnet et al., 2009). Recent evidence from *in vitro* and *in vivo* experiments shows that carbapenems including meropenem in combination with amoxicillin/clavulanate have synergistic antimycobacterial activity (Hugonnet et al., 2009; Solapure et al., 2013). Similarly, the combination of meropenem with rifampicin shows synergistic activity against not only rifampicin-sensitive *Mycobacterium tuberculosis*, but also against rifampicin-resistant strains *in vitro* (Kaushik et al., 2015). Considering the important role of rifampicin in shortening treatment duration of drug-sensitive pulmonary *tuberculosis* to 9 months and then to 6 months when combined with pyrazinamide (Controlled clinical trial, 1974; Abulfathi et al., 2019), any strategy that increases or even restores rifampicin susceptibility could improve treatment options in patients with drug-resistant *tuberculosis*.

Case reports and observational studies show that regimens containing meropenem, amoxicillin, and clavulanate has been safely used in the successful treatment of patients with MDR-/XDR-TB (Dauby et al., 2011; Payen et al., 2012; De Lorenzo et al., 2013). In addition, an influential meta-analysis of individual patient data provided evidence of better treatment outcome in MDR-TB patients receiving regimens containing carbapenems (Ahmad et al., 2018). A limitation of the meta-analysis is the observational nature of the included studies, necessitating the need for robust clinical trials to validate the findings (Ahmad et al., 2018). Diacon and colleagues recently investigated the early bactericidal activity (EBA) of meropenem administered intravenously (IV) at 2 g three times daily together with oral amoxicillin/clavulanate 500 mg/125 mg (Diacon et al., 2016). The meropenem arm resulted in a mean decline of 14-day EBA log_10_ colony-forming units (CFU) per mL of sputum of 0.11 (95% confidence interval [CI], 0.09–0.13) vs. 0.17 (95% CI, 0.15–0.19) obtained following administration of a first-line combination of rifampicin, isoniazid, pyrazinamide, and ethambutol in the same study (Diacon et al., 2016). Faropenem, an orally administered carbapenem, failed to demonstrate measurable EBA in the same study, likely owing to drug concentrations below required levels (Unpublished report, personal communication). Novel oral carbapenems are in development for *tuberculosis*. It is therefore crucial that pharmacokinetics/pharmacodynamics determinants of efficacy for carbapenems be evaluated. A population pharmacokinetics model of meropenem in patients with pulmonary *tuberculosis* is the first step in performing an integrated pharmacokinetics/pharmacodynamics analysis linking carbapenem exposure to EBA. This work aimed to develop such a model and identify covariates improving predictive performance within the COMRADE trial (NCT03174184).

## Methods

Pharma-Ethics (Ethics reference number: 170516584) and Stellenbosch University Health Research Ethics Committee (Ethics reference number: S19/01/007) approved the clinical study and this analysis, respectively.

### Study Population and Design

COMRADE is a phase 2, open-label randomized clinical trial enrolling South African men and women aged 18–65 years with sputum smear-positive pulmonary *tuberculosis*. The eligibility criteria are detailed in the Supplementary Materials. Participants with *Mycobacterium tuberculosis* strains without rifampicin-resistance conferring *rpoB* mutations were randomized into one of four study arms receiving daily treatments for 14 days: MACR2X3 received meropenem 2 g IV over 0.5 h three times daily and oral rifampicin 20 mg/kg once daily; MAC2X3 received meropenem 2 g IV over 0.5 h three times daily; MAC1X3 received meropenem 1 g IV over 0.5 h three times daily; and MAC3X1 received meropenem 3 g IV over 1 h once daily. All participants were administered oral amoxicillin/clavulanate together with each meropenem dose at doses of 500 mg/125 mg in the three times daily dose arms and at 875 mg/125 mg in the once daily dose arm. Intensive pharmacokinetics samples were collected at pre-dose and at 0.5, 1, 1.5, 2, 3, 4, 6, and 8 h post-dose at day 14 of treatment. At the end of the study, participants received Directly Observed Treatment, Short course (DOTS) to treat pulmonary *tuberculosis* as recommended in the South African National Tuberculosis Treatment Guidelines.

Participants’ data recorded included age, sex, race, weight, height, body mass index (BMI), fat-free mass (FFM), serum creatinine, creatinine clearance calculated based on Cockcroft-Gault equation (CLCR), and human immunodeficiency virus (HIV) status.

### Bioanalytical Method

Plasma meropenem concentrations were measured using validated Liquid Chromatography with Tandem Mass Spectrometry (LC-MS/MS) at FARMOVS (Pty) Ltd., South Africa (see Supplementary Materials). The quality control analysis showed acceptable reliability and reproducibility with precision and accuracy ≤15%. The lower limit of quantification (LLOQ) for meropenem was 0.5 mg/L.

### Population Pharmacokinetics Modeling

We used nonlinear mixed-effect modeling and the first-order conditional estimation with interaction (FOCE-I) method in the software NONMEM, version 7.4 for all analyses to describe the population pharmacokinetics of meropenem (Beal et al., 2004). The execution of the NONMEM control stream was implemented through Perl-speaks-NONMEM (PsN, version 4.9.0) (Lindbom et al., 2005; Keizer et al., 2013).

### Data Formatting

Data assembly, formatting, and visualizations were conducted with R (an open source statistical software, version 3.5.1) (The R Foundation 2020) and Phoenix® WinNonlin™ (version 8.1) (Phoenix WinNonlin 8.1. Ce).

### Structural and Stochastic Models

One- and two-compartment models were evaluated for the best model fit to the data. Two levels of variability were evaluated: inter-individual variability (IIV) and residual unexplained variability (representing reporting errors, assay errors, model misspecification, etc.). The IIV in pharmacokinetic parameters was assumed to be log-normally distributed. Additive, proportional, and combined error models were explored to characterize the residual unexplained variability.

### Covariate Model

Age, body weight, and CLCR are covariates previously shown to impact meropenem disposition, whereas, rifampicin is a potent inducer of drug metabolizing enzymes and transporters (Du et al., 2006; Li et al., 2006; Svensson et al., 2017; Abulfathi et al., 2019; Ehmann et al., 2019; Rapp et al., 2020). For this reason, these covariates were tested first, and those found to impact meropenem disposition were included in the model as a base for further covariate exploration using stepwise covariate model (SCM) building.

Prior to the SCM procedure, the base model with both structural and stochastic components was assessed using stepwise generalized additive modeling (GAM) implemented in Xpose to identify potential candidate empirical Bayes estimates (EBEs) and covariate relationships (Guiastrennec et al., 2017).

Finally, SCM was implemented through PsN (Lindbom et al., 2005; Keizer et al., 2013). The potential parameter-covariate relationships were tested one at a time, and the likelihood ratio test (LRT) was used to discriminate between two nested models at a statistical significance level of 5 and 1% for the forward inclusion and backward elimination procedures, respectively. The investigated covariates’ influence on meropenem pharmacokinetics parameters included those of age, height, HIV status, race, and sex on CL, and those of race and sex on V1.

### Model Selection and Evaluation

The process of model selection for nested models was based on LRT. Thus, for each additional parameter, a reduction in objective function value (OFV) of ≥3.84 corresponding to a significance level of 5% was considered statistically significant. Akaike information criterion (AIC) was used to choose between non-nested models. In addition to the goodness-of-fit statistics, the process of model selection and evaluation was guided by visual predictive checks (VPCs), prediction- and residual-based goodness-of-fit plots, and also biological plausibility, clinical relevance, and parsimony (Holford, 2008; Nguyen et al., 2017). The basic goodness-of-fit plots and VPCs were visualized with the Xpose package (version 4) and Pirana (Guiastrennec et al., 2017; Nguyen et al., 2017). Pirana was also used to manage run records.

Model validation through non-parametric bootstrapping was utilized to establish the reliability and stability of the final model (Ette et al., 2003). The bootstrapping procedure entails random sampling with replacement of each patient to form a new dataset stratified on a study arm to retain proportions of the same sample size as the original dataset. We fitted the final model to each of the 1,000 generated bootstrap datasets. The point estimates and their corresponding 95% CI were calculated for the model parameters.

## Results

**FIGURE 1:**
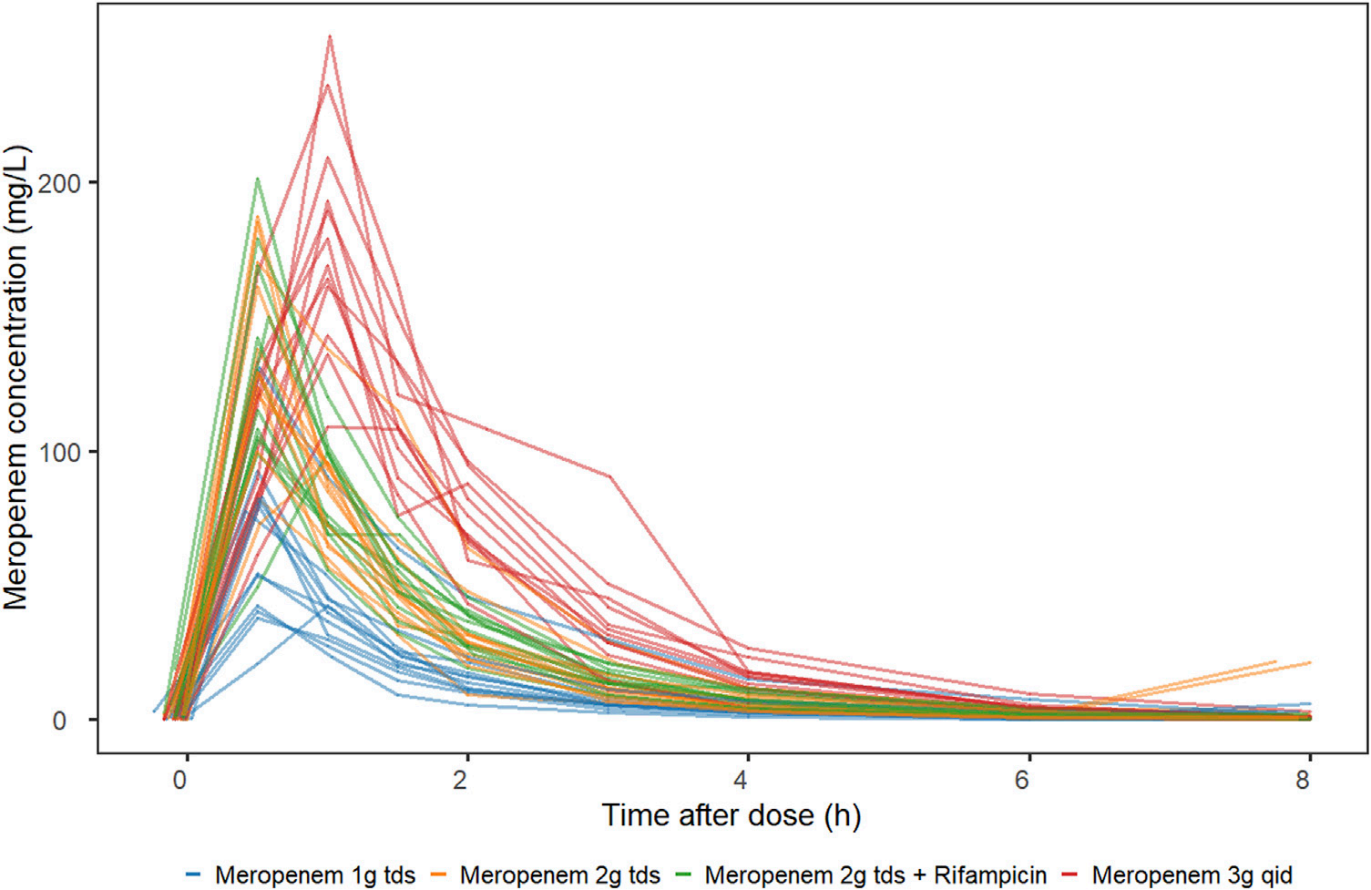
Meropenem plasma concentration-time profile stratified by study arm. MACR2X3, intravenous meropenem 2 g three times daily plus oral rifampicin 20 mg/kg once daily; MAC2X3, intravenous meropenem 2 g three times daily; MAC1X3, intravenous meropenem 1 g three times daily; MAC3X1, intravenous meropenem 3 g once daily.

Sixty participants with drug-sensitive pulmonary *tuberculosis* aged between 20 and 63 years, of whom 75% (45/60) were men, participated in the study. Of the 60 participants, 11 withdrew from the study prior to the intensive pharmacokinetic sampling visit, thus, 49 participants provided plasma samples for analysis. The demographics of the 49 participants are reported in Table 1. Of the 441 concentration observations available, 404 were included in the analysis. Whereas 34 plasma samples were below the quantification limit (BQL), three samples (one at pre-dose and two at 8 h after dose) were excluded with the motivation that the concentrations were at least 10-fold higher than expected, and their conditional weighted residuals (CWRES) ≥4. Figure 1 displays the individual meropenem concentration-time profiles per study arm.

**TABLE 1:** Characteristics of patients who participated in pharmacokinetic sampling.

Characteristics	MACR2X3 (*n* = 12)	MAC2X3 (*n* = 13)	MAC1X3 (*n* = 12)	MAC3X1 (*n* = 12)	Overall (*n* = 49)
Age (years)					
Median (Q1, Q3)	32.3 (27.6, 40.2)	36.5 (33.2, 45.4)	40.9 (28.6, 45.8)	34.0 (28.2, 39.1)	36.0 (28.6, 45.4)
Max-min	21.1–58.6	23.1–61.2	20.0–62.7	20.3–55.6	20.0–62.7
**Sex**					
Female	3 (25.0%)	6 (46.2%)	2 (16.7%)	1 (8.3%)	12 (24.5%)
**Race**					
Black	2 (16.7%)	2 (15.4%)	5 (41.7%)	7 (58.3%)	16 (32.7%)
Mixed Asian ancestry	10 (82.3%)	11 (84.6%)	7 (58.3%)	5 (41.7%)	33 (67.3%)
**HIV status**					
Positive	1 (8.3%)	3 (23.1%)	3 (25.0%)	4 (33.3%)	11 (22.4%)
**Weight (kg)**					
Median (Q1, Q3)	52.3 (48.2, 55.9)	50.3 (48.3, 55.5)	55.2 (51.6, 62.1)	49.6 (45.8, 56.8)	52.7 (47.5, 57.1)
Max-min	39.3–62.4	40.3–65.9	45.1–65.5	43.0–76.3	39.3–76.3
**Height (m)**					
Median (Q1, Q3)	1.65 (1.60, 1.68)	1.62 (1.57, 1.71)	1.73 (1.67, 1.7)	1.66 (1.62, 1.69)	1.66 (1.60, 1.71)
Max-min	1.54–1.76	1.54–1.82	1.58–1.76	1.59–1.73	1.54–1.82
**Creatinine clearance (ml/min)**					
Median (Q1, Q3)	126 (90.3, 145)	109 (83.3, 139)	98.6 (94.1, 129)	112 (99.5, 127)	115 (94.3, 137)
Max-min	76.7–203	57.7–173	61.9–187	93.9–185	57.7–203

MACR2X3, intravenous meropenem 2 g every 8 h plus oral rifampicin 20 mg/kg once daily; MAC2X3, intravenous meropenem 2 g every 8 h; MAC1X3, intravenous meropenem 1 g every 8 h; MAC3X1, intravenous meropenem 3 g once daily; HIV, human immunodeficiency virus; Q1, lower quartile; Q3, upper quartile; Min, minimum; Max, maximum.

**FIGURE 2:**
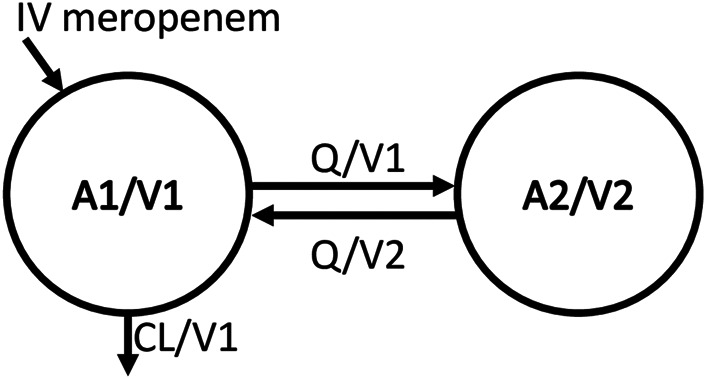
Structural model schema. Meropenem amount in the central compartment (A1), central volume of distribution (V1), intercompartmental clearance (Q), meropenem amount in the peripheral compartment (A2), peripheral volume of distribution (V2), total plasma clearance (CL), meropenem concentration in the central compartment (A1/V1), meropenem concentration in the peripheral compartment (A2/V2), elimination rate constant is CL/V1, transfer rate constant from central to peripheral compartment (Q/V1), and transfer rate constant from peripheral to central compartment (Q/V2).

The meropenem concentration-time data were fitted best with a two-compartment model (Figure 2). Table 2 provides the estimated typical values of the structural pharmacokinetic parameters with low uncertainty in parameter estimates ranging from 3.8 to 27.5%.

**TABLE 2:** Meropenem population pharmacokinetic model parameters.

Parameter	Population estimate (%RSE^a^)	Bootstrap median (95% CI)
**Structural model parameter**		
CL (L/h/70 kg)	11.8 (4.9)	11.9 (10.5–12.8)
V1 (L/70 kg)	14.2 (3.8)	14.6 (13.4–16.4)
Q (L/h/70 kg)	3.26 (27.5)	3.15 (0.777–4.84)
V2 (L/70 kg)	3.12 (10.8)	3.17 (1.54–78.4)
**Inter-individual variability (IIV) as %CV** **^b^**		
IIV CL	20 (15.5)	19.3 (13.7–25.4)
IIV V1	13.1 (35.4)	12.7 (0.131–21.2)
IIV V2	106 (30.7)	111 (0.868–710)
**Residual variability**		
Proportional residual error (%)	0.178 (14.8)	0.178 (0.127–0.229)
Additive residual error (mg/L)	1.16 (19.6)	1.13 (0.388–1.54)
**Covariate**		
Creatinine clearance on CL	0.416 (30.5)	0.403 (0.203–0.704)

a Relative standard error (%RSE) was calculated as the standard error from the covariance step/population estimate.

b Coefficient of variation (%CV) for IIV was calculated as (SQRT (EXP(OMEGA)-1)*100.

Confidence interval (CI), clearance from the central compartment (CL), central volume of distribution (V1), intercompartmental clearance (Q), and peripheral volume of distribution (V2). The bootstrap median and 95% CI were calculated from fitting the final model to the 1,000 bootstrap datasets. TVCL = THETA (1)*((WTKG/70)**0.75)*((CLCR*70/WTKG)/115)**THETA (7); TVCL is the meropenem clearance in the typical individual. TVV1 = THETA (2)*WTKG/70; TVV1 is the meropenem volume of distribution in the central compartment in the typical individual. TVQ = THETA (3)*((WTKG/70)**0.75); TVQ is the meropenem inter-compartmental clearance in the typical individual. TVV2 = THETA (4)*WTKG/70; TVV2 is the meropenem volume of distribution in the peripheral compartment in the typical individual.

We estimated a relatively low IIV in CL and V1 with a coefficient of variation (CV) of 20 and 13.1%, respectively. No significant variability could be detected in Q while for V2 the variability between individuals was high (CV of 106%).

A combined additive and proportional error model was used to quantify the residual unexplained variability (Table 2).

The addition of allometric scaling with body weight on disposition parameters, normalized to 70 kg with fixed theoretical exponents of 1 for volume of distribution and 0.75 for clearance, resulted in a 22.2 points OFV reduction (Anderson and Holford, 2009). A further 14.4 points reduction in OFV occurred with the inclusion of size-standardized CLCR normalized to the median population value of 115 ml/min. Conversely, both age and rifampicin had an insignificant impact on meropenem CL.

Following the SCM’s three forward selection and two backward elimination procedures, none of the other parameter-covariate relationships met the criteria for inclusion in the full final model.

**FIGURE 3:**
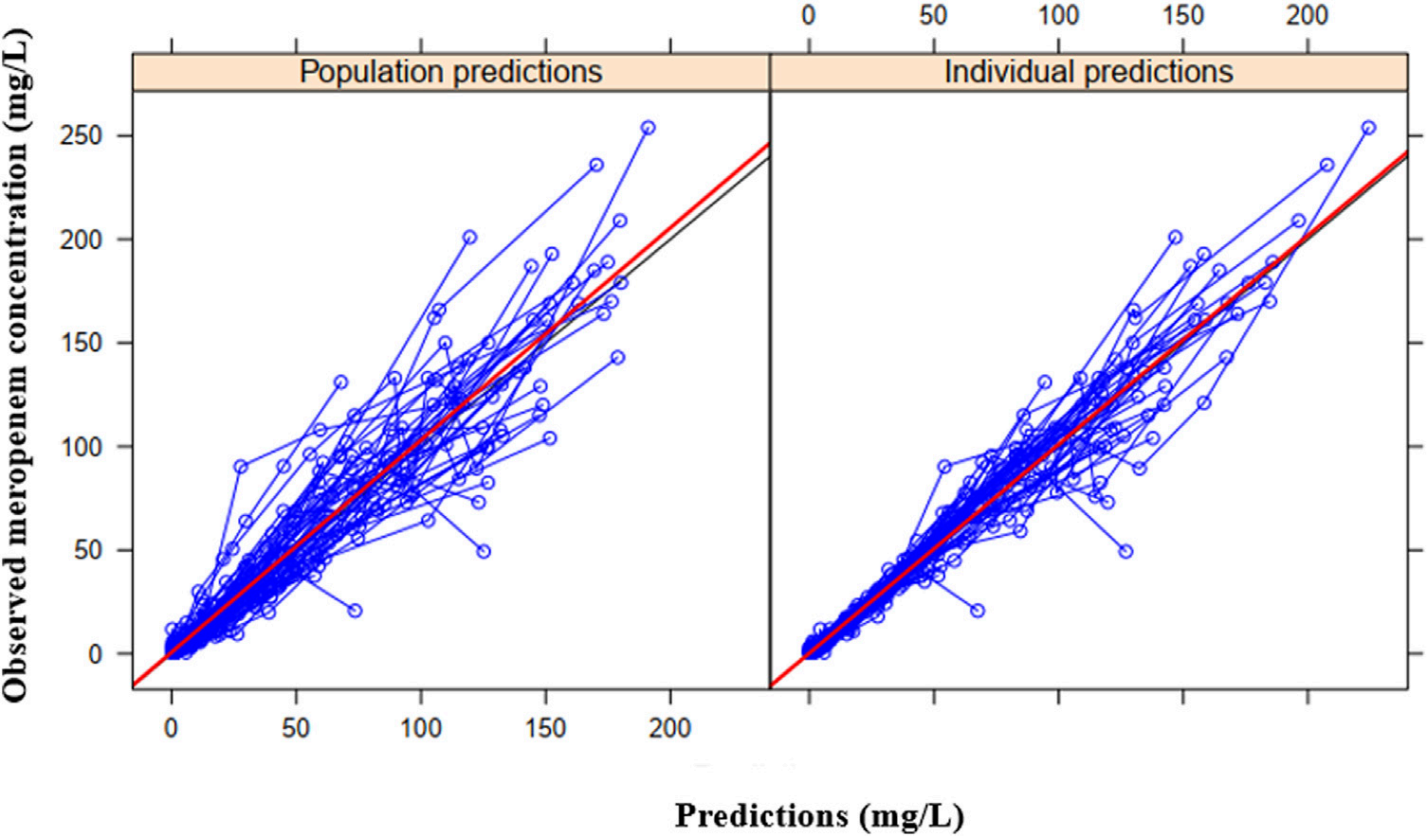
Basic goodness-of-fit plots of the final model showing the observed meropenem concentration vs. the individual predicted concentration **(right)** or population predicted concentration **(left)**. The observed and predicted concentrations are from the 49 individuals in the study.

**FIGURE 4:**
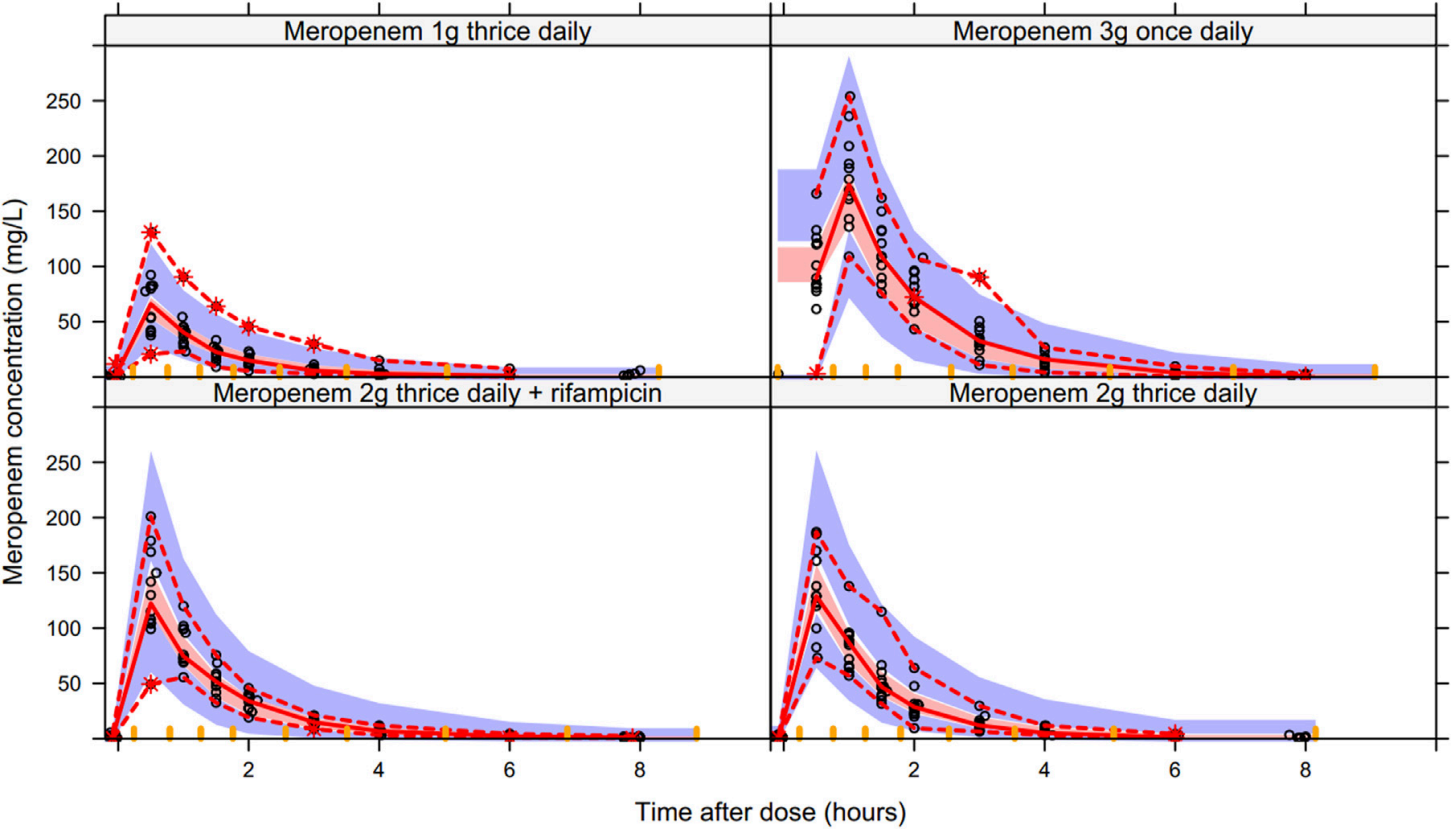
Visual predictive check of the final model stratified by study arms. The dashed red lines represent the 97.5th and 2.5th percentiles of the observed meropenem concentration data (open black circles), the solid red line connects the median (50th percentile) of the observed data (*n* = 49). The blue shaded areas represent 95% confidence intervals of the 97.5th and 2.5th percentiles of the predicted simulated data (*n* = 1,000), whereas the red shaded area represents 95% confidence interval of the median (50th percentile) of the predicted simulated data.

The final model provides good fit to the observed population distribution of concentration-time data (Figure 3), and the observed individual concentration-time profiles (Supplementary Figure S1). Supplementary Figures S2A,B display plots of residual-based diagnostics, whereas, Figure 4 displays the VPCs of the final model.

Model validation through non-parametric bootstrap procedure demonstrates the final model’s robustness in describing meropenem pharmacokinetics (Table 2).

## Discussion

To our knowledge, we describe for the first time, the population pharmacokinetics of meropenem in patients with *tuberculosis*. A two-compartment model fit the data best. We estimated with good precision the typical values of CL, Q, V1, and V2. The IIV in V2 was high, but low for CL and V1. The structural, stochastic, and covariate parameter estimates of the typical individual obtained from NONMEM analysis fell within the 95% CI of the non-parametric bootstrapping procedure, an indication of the model robustness in predicting meropenem concentrations (Table 2). However, the uncertainty in the estimate of IIV on V2 was high (Table 2). The difficulty in estimating the IIV on V2 did not affect the model’s purpose of describing meropenem population pharmacokinetics. Further, rifampicin did not affect meropenem CL in the current study. While this is not surprising, because meropenem is predominantly excreted unchanged in urine, rifampicin, a potent inducer of both metabolizing enzymes and transporters, could theoretically increase meropenem CL by inducing renal drug transporters. This provides reassurance that these two drugs can be used together, as needed, without need for dose adjustment to mitigate a drug interaction.

In the current study, meropenem CL of 11.8 L/h in a 70 kg individual with a CLCR of 115 ml/min confirms previous reports that renal elimination of meropenem involves both the processes of glomerular filtration and tubular secretion (Rapp et al., 2020; Meronem500mg - Summar). Meropenem is a polar carbapenem that distributes into extracellular fluid, with approximately 70% of a dose excreted unchanged in urine (Rapp et al., 2020; Meronem500mg - Summar). It is biologically plausible to expect meropenem clearance to change with body weight and renal function. To this end, the inclusion of allometric scaling with body weight on disposition parameters resulted in a drop in OFV by 22.2 points, and thus, improved the model fit (Anderson and Holford, 2009). Allometric scaling with centering at 70 kg was done to allow the comparison of the disposition parameters with results of other studies in adults or children. For example, Rapp and colleagues reported the typical value of clearance of 6.82 L/h normalized to a 70 kg adult (Rapp et al., 2020). Reasons for the lower clearance in this population of critically ill children compared to 11.8 L/h in the current study could include renal impairment and/or maturation in organ function in the very young.

Other investigators sequentially evaluated the covariate effect of body weight on disposition parameters and found a significant impact on V1 only (Li et al., 2006). This is in contrast with our approach, and that of others (Rapp et al., 2020), in which body weight was included as the allometric size-descriptor simultaneously on all disposition parameters. This is based on the understanding that volumes increase linearly with body weight (fixed theoretical exponent of 1) while clearance increases following a power function (fixed theoretical exponent of 0.75) (Anderson and Holford, 2009).

Because of the polar nature of meropenem, it is reasonable to expect a better model fit when allometric scaling is with FFM rather than total body weight. On the contrary, the model with FFM resulted in a lesser (20.4 points) OFV reduction, than that with body weight (22.2 points). For this reason and that of parsimony, we chose to keep the model with total body weight. The finding is not entirely surprising given the data: the median weight in the current study population is 52.7 kg (range, 39.3–76.3), and no patient was obese. The model with FFM could be more useful when describing patients with extreme body weights (Al-Sallami et al., 2015).

We found creatinine clearance to account for some variability in meropenem clearance between individuals and to provide an improvement in goodness-of-fit statistics. The covariate effect co-efficient of size-standardized creatinine clearance on CL is 0.416 (95% CI of 0.171–0.661) in the current study and is similar to 0.62 (95% CI of 0.34–0.83) reported by Li and colleagues (Li et al., 2006). Other investigators documented similar results in children (Du et al., 2006; Rapp et al., 2020). The clinical implication of the estimated effect of creatinine clearance on CL is that in a 70 kg patient with severe renal impairment (CLCR of 5–30 ml/min), about a 40–70% reduction in meropenem doses would be required. Compared to other studies (Du et al., 2006; Li et al., 2006), the relatively low IIV in CL and V1 in the current study might be explained by the homogeneous patient population.

Although, few investigators reported the significant effect of age on meropenem CL, no such effect was seen in the current study (Du et al., 2006; Li et al., 2006). It should be noted however, that the age range in our study was 20–63 years, but 18–93 years in the study by Li and colleagues (Li et al., 2006). Whereas the study by Du and colleagues enrolled children aged 0.08–17.3 years (Du et al., 2006). The significant impact of age on drug clearance in children could be explained in part by the effect on drug elimination of size and maturation of organ function (Holford et al., 2013).

This study has limitations. Firstly, the model was developed in adult *tuberculosis* patients and cannot be extrapolated to children. Secondly, the sample size might not provide enough power to pick up covariate relations with weak effects or those occurring very rarely. However, such covariate effects are of limited clinical importance.

## Conclusion

A two-compartment population pharmacokinetics model described the pharmacokinetics of meropenem well with good precision in parameter estimates. The addition of both allometric scaling with body weight on disposition parameters and creatinine clearance on meropenem clearance increased the model’s predictive performance. Rifampicin exposure did not influence meropenem parameters. The model will be used for integrated pharmacokinetics/pharmacodynamics analysis linking meropenem exposure to early bactericidal activity.

## Data Availability

The corresponding author can be contacted for consideration of a reasonable request of the data supporting the conclusion of this study.
