# Supplementary material for: The Population Pharmacokinetics of Meropenem in Adult Patients With Rifampicin-Sensitive Pulmonary Tuberculosis
Source: Front Pharmacol. 2021 Jun 29;12:637618. doi: 10.3389/fphar.2021.637618 (PMC8275874; doi:10.3389/fphar.2021.637618)
Supplement: Supplementary file 1 [file DataSheet1.docx]

**The population pharmacokinetics of meropenem in adult patients with rifampicin-sensitive pulmonary tuberculosis**

Ahmed A. Abulfathi,^1,2^ Veronique de Jager,^3^ Elana van Brakel,^3^ Helmuth Reuter,^1^ Nikhil Gupte,^4^ Naadira Vanker,^3^ Grace L. Barnes,^4^ Eric Nuermberger,^4^ Susan E. Dorman,^5^ Andreas H. Diacon,^3,6^ Kelly E. Dooley,^7^ Elin M. Svensson.^8,9^

(1) Division of Clinical Pharmacology, Department of Medicine, Faculty of Medicine and Health Sciences, Stellenbosch University, Cape Town, South Africa.

(2) Department of Clinical Pharmacology and Therapeutics, College of Medical Sciences, University of Maiduguri, Maiduguri, Nigeria.

(3) Task Applied Science, Bellville, South Africa.

(4) Department of Medicine, Center for Tuberculosis Research Johns Hopkins University Baltimore, Maryland, United States of America.

(5) Department of Medicine, Medical University of South Carolina, Charleston, SC, United States of America.

(6) Department of Medicine, Faculty of Medicine and Health Sciences, Stellenbosch University, Cape Town, South Africa.

(7) Divisions of Clinical Pharmacology and Infectious Diseases, Department of Medicine, Johns Hopkins University Center for Tuberculosis Research, Baltimore, United States of America.

(8) Department of Pharmaceutical Biosciences, Uppsala University, Uppsala, Sweden.

(9) Department of Pharmacy, Radboud Institute for Health Sciences, Radboud University Medical Center, Nijmegen, the Netherlands.

**Corresponding author**

Prof Andreas H. Diacon

Department of Medicine,

Faculty of Medicine and Health Sciences,

Stellenbosch University,

Cape Town, South Africa.

ahd@sun.ac.za

Supplementary materials:

Methods

Figure S1: Meropenem population pharmacokinetics model fit to individual data

Figure S2a: CWRES versus population prediction

FigureS2b: CWRES versus time after dose

NONMEM control stream of the final model

**Methods**

**Eligibility Criteria**

Inclusion criteria for participation in this study

- New or recurrent pulmonary tuberculosis (TB) with one or both of the following:
  - Sputum positive for acid-fast bacilli on direct microscopy of at least grade 1+ (International Union Against Tuberculosis and Lung Disease [IUATLD] scale) on at least one pre-treatment sputum sample.
  - Sputum positive for M. tuberculosis by Xpert MTB/RIF testing, semiquantitative result of “medium” or “high” on at least pre-treatment sputum sample.
- Age ≥18 and ≤65 years at screening.
- Ability and willingness to provide informed consent.
- Body weight 40 kg to 90 kg, inclusive.
- Laboratory values obtained within 30 days prior to or at study screening:
  - Absolute neutrophil count (ANC) ≥750 cells/mm^3^.
  - Hemoglobin ≥7.0 g/dL.
  - Platelet count ≥50,000/ mm^3^.
  - Serum aspartate aminotransferase (AST) and alanine aminotransferase (ALT) ≤3 X upper limit of normal (ULN).
  - Serum total bilirubin ≤2.5 X ULN.
  - Serum creatinine <1.5 X ULN.
- Human immune-deficiency virus (HIV) infection must be documented as either absent or present
- For HIV-positive participants, only: CD4+ cell count of ≥100 cells/mm^3^, performed within 30 days prior to or at study screening.
- For females of reproductive potential, negative serum or urine pregnancy test within 7 days prior to study screening. Female participants who are engaging in sexual activity that could lead to pregnancy must agree to use one reliable non-hormonal method of contraception (condoms or an intra-uterine contraceptive device), or another method (diaphragm or cervical cap) if it is approved by the national regulatory authority and used according to package insert, while receiving study medications.
- Willingness to be hospitalized for a minimum of 16 consecutive days.
- Ability to produce an overnight sputum sample of sufficient quality and quantity. As a guideline, this should be 10 mL or more during a 16-hour collection period.
- Xpert MTB/RIF result performed on sputum within 14 days prior to or at study screening that shows either “Rifampicin resistance detected” or “Rifampicin resistance not detected”.

Exclusion criteria

- Treatment with any drug active against *M. tuberculosis* within the 3 months prior to study screening.
- Breast-feeding.
- Known allergy or sensitivity to any of the study drugs.
- Participants receiving valproate sodium or probenecid.
- Karnofsky score <60 or poor general condition such that, in the opinion of the investigator at screening, any delay in initiation of definitive TB treatment cannot be tolerated.
- Known current neurological TB or seizure disorder.
- Any condition as determined by physical examination, medical history, laboratory data, or chest x-ray which, in the opinion of the investigator, would interfere with safety or endpoint assessments in the study.

**Bioanalytical method**

Meropenem was quantified by a validated analytical method using Liquid Chromatography with Tandem Mass Spectrometry (LC-MS/MS). The validated method was developed to simultaneously quantify meropenem and ertapenem in human dipotassium ethylenediaminetetraacetic acid (K_2_EDTA) plasma over the range of 0.5-256 μg/mL.

The analytes were extracted from the biological matrix (i.e., plasma) using protein precipitation with methanol. This was followed by dilution of the supernatant with a mixture of acetonitrile and formic acid solution.

The liquid chromatographic separation was with Phenomenex® Kinetex XB-C18, 150 x 4.6 mm, 5 μm analytical column. Mobile phases: mobile phase A (formic acid solution) and B (acetonitrile) were delivered using a gradient flow. The autosampler, equipped with a 96-well tray, was used to inject 2 μL of each sample onto the column, at a temperature of approximately 5 ºC. Meropenem retention time was ~1.44 minutes.

Mass spectrometer, Sciex API4000 coupled to Watson LIMS™ software version 7.4.2 and Analyst® software version 1.6.2 was used. Ionization mode was with electrospray Ionization (ESI) in positive mode. Meropenem protonated precursor ion with m/z 384.0 and product ion with m/z 141.0. The internal standard working solution with a concentration of ~12 μg/mL meropenem-d6 in methanol was prepared in a polypropylene container by dissolving the reference substance directly in the methanol. The internal standard working solution was added to each sample (excluding blank samples). Calibration standards (STDs) in human K_2_EDTA plasma and quality control samples (QCs) in human K_2_EDTA plasma and in stabilized human lithium heparin (LH) plasma, were prepared gravimetrically in human plasma.

The validation process comprised of three accuracy and precision validation runs during which the accuracy and precision of the method was established and evaluated against acceptance criteria as defined by the regulatory guidelines. The meropenem regression model used was: log-log linear calibration curve (log y = a logx + b. The Response Type was peak area ratio. Absolute recovery (extraction efficiency) for meropenem was 90.4% (mean % coefficient of variation [%CV] = 4.7), whereas, the relative recovery was 0.903 (mean %CV = 4.0). The average Signal-to-Noise Ratio at lower limit of quantification (LLOQ) was 54.4. No inherent carry-over was detected in the blank samples. In addition, no interfering peaks from endogenous and other matrix components were observed at the retention times of meropenem or the internal standard.

Meropenem was accurately quantified in the presence of commonly used over-the-counter drugs (paracetamol, ibuprofen, cyclizine, cetirizine, pseudoephedrine, codeine and diclofenac). A multi-component analysis confirmed that meropenem can be accurately determined in the presence of amoxicillin, clavulanic acid, rifampicin and each other. The variability of the internal standard-normalized matrix factor (IS-MF) was <15% at both low and high analyte concentrations, indicating that the analysis is reproducible in the various matrices.

Dilution integrity: the following dilutions were successfully validated;

1. 2-fold dilution: K_2_EDTA plasma samples diluted with K_2_EDTA plasma.
2. 2-fold dilution: stabilized LH plasma samples diluted with stabilized LH plasma.
3. 5-fold dilution: stabilized LH plasma samples diluted with K_2_EDTA plasma.
4. 15-fold dilution: K_2_EDTA plasma samples diluted with K_2_EDTA plasma.

Plasma samples with meropenem concentration above the upper limit of quantification can be analyzed by applying 2-fold, 5-fold and 15-fold dilutions.

Finally, the 3 consecutive accuracy and precision validation runs met the acceptance criteria:

1. The between-run accuracy calculated over all 3 consecutive validation runs (expressed as %Bias) must be within 15% over the range and within 20% of at the LLOQ, and
2. The between-run precision calculated over all 3 consecutive validation runs (expressed as %CV) must be ≤15% (20% at the LLOQ).

**Figure S1.** Meropenem population pharmacokinetics model fit to individual data


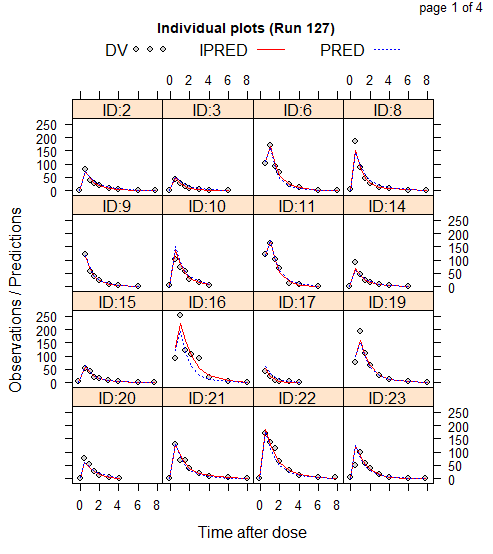


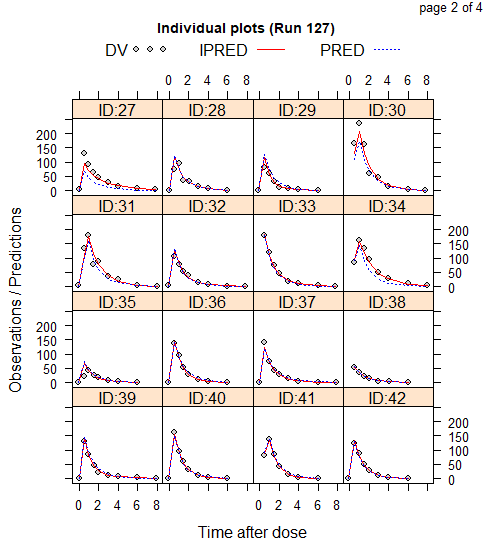


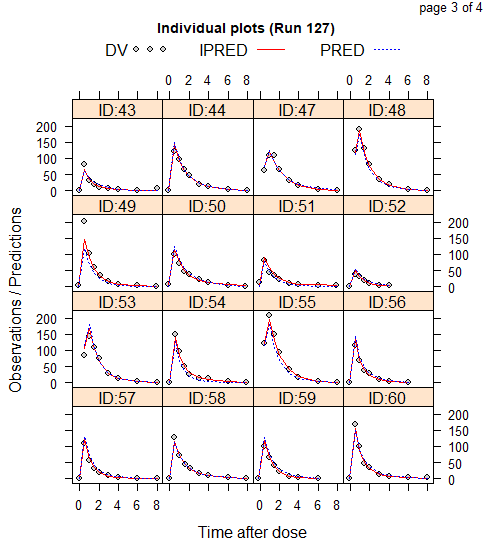


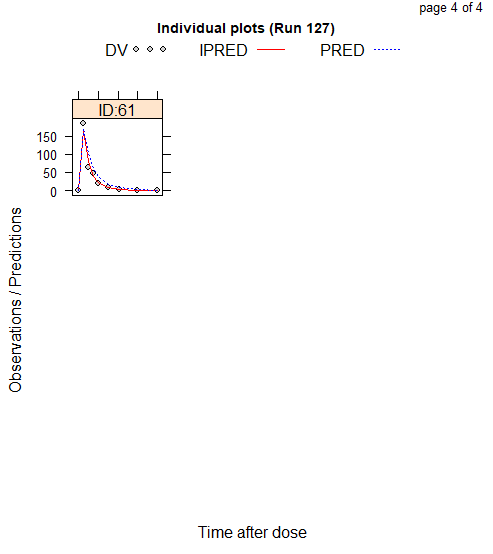


DV, observed meropenem concentration (mg/L); IPRED, individual prediction (mg/L); PRED, population prediction (mg/L).

**Figure S2a.** CWRES versus population prediction in mg/L


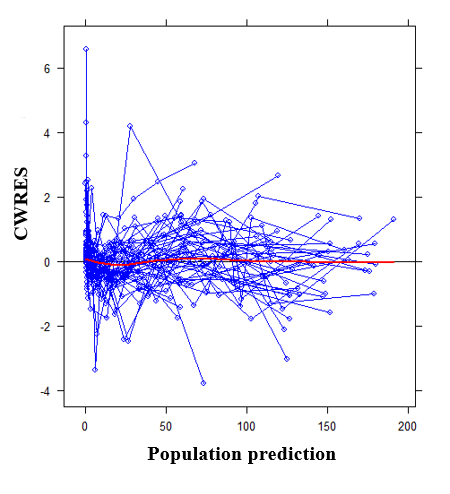


CWRES, conditional weighted residuals. The blue open circles are the observed concentrations. The solid black horizontal line is the zero line. If the predictions were 100% perfect, all the blue open circles will fall on the solid black line. The solid red line is the trend line.

**Figure S2b.** CWRES versus time after dose


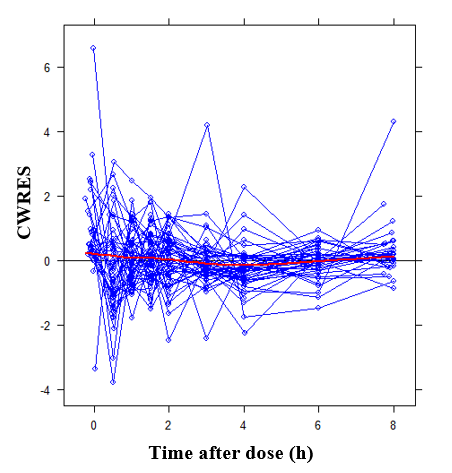


CWRES, conditional weighted residuals. The blue open circles are the observed concentrations. The solid black horizontal line is the zero line. If the predictions were 100% perfect, all the blue open circles will fall on the solid black line. The solid red line is the trend line.

NONMEM control stream of the final model

$PROBLEM Meropenem population pharmacokinetics modeling in patients with pulmonary tuberculosis.

$INPUT ID USUBJID DAT2=DROP TIME TAD TAD2 SAMPT=DROP DURHR AMT

MDV EVID DV BLQP CMT RATE SS=DROP DOMAIN=DROP VISIT EXCL

EXCL2 PAGE CLDV PCSP AGE SEXF RACEB HTM WTKG BMI FFM LBW

HIVP RIFGPS RIF1 AMXCLV G2ARM DOSE CREAT CLCR SDAY

SUBJECT=DROP STAD=DROP LASTDOSE=DROP

$DATA Datasetxx.csv IGNORE=@ IGNORE=(EXCL2.EQ.1) IGNORE=(EXCL2.EQ.4) IGNORE=(EXCL2.EQ.5)

$SUBROUTINE ADVAN13 TOL=9

$MODEL COMP (COMP1) ;Central compartment

COMP (COMP2) ;Peripheral compartment

$PK

D1=DURHR

TVCL = THETA(1)*((WTKG/70)**0.75)*(((CLCR*70/WTKG)/115)**THETA(7)) ;TVCL is the clearance of the typical individual in the population

;+ allometric scaling with body weight centered at 70 kg ;+ Covariate effect of size-standardized creatinine clearance ;centered at 115 mL/min

TVV1 = THETA(2)*WTKG/70 ;TVV1 is the central compartment's volume of distribution for the ;typical individual in the population plus

;allometric scaling with body weight centered at 70 kg

TVQ = THETA(3)*((WTKG/70)**0.75) ;TVQ is the intercompartmental clearance of the typical individual ;in the population plus allometric scaling with body weight ;centered at 70 kg

TVV2 = THETA(4)*WTKG/70 ;TVV2 is the peripheral compartment's volume of distribution for ; the typical individual in the population plus allometric scaling ;with body weight centered at 70 kg

CL = TVCL*EXP(ETA(1)) ;Individual clearance, and inter-individual variability (IIV)

V1 = TVV1*EXP(ETA(2)) ;Individual volume of distribution of the central compartment, and IIV

Q = TVQ*EXP(ETA(3)) ;Individual intercompartmental clearance, and IIV

V2 = TVV2*EXP(ETA(4)) ;Individual volume of distribution of the peripheral compartment, and IIV

$DES

CENTRAL_DES = A(1)

PERIPHERAL_DES = A(2)

CC_DES = (CENTRAL_DES/V1)

DADT(1) = (((-(Q)*(A(1)/V1))+(Q*(A(2)/V2))-(CL*(A(1)/V1))))

DADT(2) = ((Q*(A(1)/V1))-(Q*(A(2)/V2)))

$ERROR ;Residual error model

CENTRAL = A(1) ;Amount in central compartment

PERIPHERAL = A(2) ;Amount in peripheral compartment

CC = (CENTRAL/V1) ;Concentration in the central compartment

IPRED = CC ;Individual prediction

IRES = DV - IPRED

W = SQRT((THETA(5)*IPRED)**2 + THETA(6)**2) ;For proportional and additive errors

IWRES = IRES/W

Y = IPRED+W*EPS(1)

$THETA

(0.0,11.8026) ; 1. TVCL

(0.0,14.2024) ; 2. TVV1

(0.0,3.26491) ; 3. TVQ

(0.0,3.11527) ; 4. TVV2

(0,0.178409) ; 5. Proportional error

(0,1.15559) ; 6. Additive error

0.415838 ; 7. Covariate effect of CLCR

$OMEGA

0.0392918 ; 1. IIV_CL

0.0170819 ; 2. IIV_V1

0 FIX ; 3. IIV_Q

0.753935 ; 4. IIV_V2

$SIGMA 1.0 FIX

$ESTIMATION METHOD=COND INTER NSIG=3 SIGL=9 MAXEVALS=9999 PRINT=10

NOABORT MSFO=xx.msf

$COVARIANCE

$TABLE ID USUBJID TAD MDV EVID AMT RATE AGE WTKG CLCR HTM BMI FFM

LBW RACEB SEXF HIVP G2ARM DOSE BLQP PRED IPRED RES IRES

IWRES WRES CWRES CIPRED CIRES CIWRES Y DV NOAPPEND NOPRINT

ONEHEADER FILE=sdtabxx

$TABLE ID CL V1 Q V2 ETA(1) ETA(2) ETA(3) ETA(4) NOAPPEND NOPRINT

ONEHEADER FILE=patabxx

$TABLE ID AGE WTKG FFM LBW CLCR HTM BMI NOAPPEND NOPRINT

ONEHEADER FILE=cotabxx

$TABLE ID RACEB SEXF HIVP G2ARM RIF1 NOAPPEND NOPRINT ONEHEADER

FILE=catabxx
